# Incentivisation practices and their influence on physicians’ prescriptions: A qualitative analysis of practice and policy in Pakistan

**DOI:** 10.1371/journal.pgph.0001890

**Published:** 2023-06-29

**Authors:** Mishal Khan, Afifah Rahman-Shepherd, Muhammad Naveed Noor, Sabeen Sharif, Meherunissa Hamid, Wafa Aftab, Afshan Khurshid Isani, Robyna Irshad Khan, Rumina Hasan, Sadia Shakoor, Sameen Siddiqi

**Affiliations:** 1 Department of Global Health, Faculty of Public Health and Policy, London School of Hygiene and Tropical Medicine, London, United Kingdom; 2 Department of Pathology and Laboratory Medicine, Aga Khan University, Karachi, Pakistan; 3 Department of Global Public Health and Primary Care, University of Bergen, Bergen, Norway; 4 Department of Health, Government of Sindh, Karachi, Pakistan; 5 Department of Anaesthesiology, Aga Khan University, Karachi, Pakistan; PLOS: Public Library of Science, UNITED STATES

## Abstract

Focus on profit-generating enterprise in healthcare can create conflicts of interest that adversely impact prescribing and pricing of medicines. Although a global challenge, addressing the impacts on quality of care is particularly difficult in countries where the pharmaceutical industry and physician lobby is strong relative to regulatory institutions. Our study characterises the range of incentives exchanged between the pharmaceutical industry and physicians, and investigates the differences between incentivisation practices and policies in Pakistan. In this mixed methods study, we first thematically analysed semi-structured interviews with 28 purposively selected for-profit primary-care physicians and 13 medical sales representatives from pharmaceutical companies working across Pakistan’s largest city, Karachi. We then conducted a content analysis of policies on ethical practice issued by two regulatory bodies responsible in Pakistan, and the World Health Organization. This enabled a systematic comparison of incentivisation practices with what is considered ‘prohibitive’ or ‘permissive’ in policy. Our findings demonstrate that incentivisation of physicians to meet pharmaceutical sales targets is the norm, and that both parties play in the symbiotic physician-pharma incentivisation dynamics. Further, we were able to categorise the types of incentive exchanged into one of five categories: financial, material, professional or educational, social or recreational, and familial. Our comparison of incentivisation practices with policies revealed three reasons for such widespread incentivisation linked to sales targets: first, some clear policies were being ignored by physicians; second, there are ambiguous or contradictory policies with respect to specific incentive types; and third, numerous incentive types are unaddressed by existing policies, such as pharmaceutical companies paying for private clinic renovations. There is a need for policies to be clarified and updated, and to build buy-in for policy enforcement from pharmaceutical companies and physicians, such that transgressions on target-driven prescribing are seen to be unethical.

## Introduction

There are differing views among policymakers and researchers on the optimal role of the private health sector in contributing to healthcare, including the role of both for-profit healthcare providers and the pharmaceutical industry [1]. On the one hand, the private, for-profit health sector has expanded access to healthcare products and services that are needed [2]. On the other, there are concerns about increases in health-related costs, the growing reliance on the private sector to provide healthcare services rather than the public sector, and challenges to effectively regulate this sector [2]. It is clear that profit-generating business models of healthcare providers and the pharmaceutical industry can create conflict of interests (COI) that may compromise the quality of care provided to patients such that the prescribed medicines are unnecessary or more costly than equivalent alternatives [3–6]. Indeed, in 1993, in relation to rational drug use, the World Health Organization (WHO) acknowledged that there is an ‘inherent COI between the legitimate business goals of manufacturers and the social, medical and economic needs of providers and the public’ [7]. Downstream impacts of these COI include excess costs to patients, undue adverse effects, and drug resistance [8–11].

In the context of medical care, we define COI as a situation in which the objectivity of an individual or institution’s professional judgement is compromised, or could be compromised, by a secondary interest [6]. A COI can exist without the individual or institution consciously or tangibly influenced. This distinguishes COI from other practices that can be more narrowly defined as corruption, whereby entrusted power is consciously abused for private gain, and makes COI more challenging to investigate in any given setting [12]. Evidence from a range of settings shows that COI can lead to physician-induced demand for healthcare in which physicians seek to maximise their profits by influencing patients to use healthcare services or products against what would be in the patient’s best interest [13–15]. This can result in the prescription of procedures and medicines that are inappropriate, unnecessary, and/or more costly than available alternatives [7]. The pharmaceutical industry is a central player in this dynamic.

In 2018, the WHO estimated the global pharmaceutical market was worth USD$1.4 trillion per annum [16], indicating the substantial economic power this industry has to leverage to influence policymaking and regulation at both global and national levels. Worldwide, pharmaceutical companies promote their products via medical sales representatives (MSR), who use an extensive array of strategies to influence—consciously and subconsciously—physicians decision-making to boost sales of their companies’ products and hit specific sales targets [16–18]. The pharmaceutical industry invests substantial resources in inducing demand for medicines by both physicians and patients: of the top 100 pharmaceutical companies by sales in 2015, 64% spent twice the amount on marketing and sales than on research and development [16].

Addressing COI is particularly difficult in countries where the pharmaceutical industry is booming yet healthcare governance and regulatory bodies are still in their infancy, developing mandates alongside complex, rapidly evolving pharma-physician dynamics [6, 13]. Evidence suggests that the challenges to tackling inappropriate relationships between pharmaceutical companies and physicians range from regulators being under-resourced or ‘captured’ by the industry to a lack of clarity on rules, such as disclosures by physicians receiving gifts or money from pharmaceuticals, and gaps in medical training and ethics education [6, 13, 19]. Traditional forms of ‘hard regulation’ to address physician-induced demand, such as imposing standards with sanctions or penalties in the case of non-compliance, are reported to have had limited impact in several low- and middle-income countries [20–23]. This is partly because hard regulations assume a top-down relationship between the regulator and the regulated entity, such as physicians or pharmaceutical companies, and often insufficiently account for the power of these two groups relative to the regulator. Overall, the development and enforcement of regulations is weak, and regulations remain underdeveloped and outdated [21, 24, 25]. Understanding views, practices and relationships of pharmaceutical industry stakeholders and physicians is therefore essential for the co-development of strategies to build the social and political support required for reforms to be successfully introduced by regulators [26].

### Pharma-physician dynamics in the Pakistan context

Pakistan is the fifth most populous country in the world with a population of approximately 216.6 million people. Out-of-pocket spending on healthcare has been declining since the early 2000s from around 78% to 56% by 2018 according to World Bank data, although studies suggest that between 57% and 80% of health service utilization still occurs in the private sector [27]. Meanwhile, the value of the pharmaceutical industry has doubled over the past decade and more than 620 pharmaceutical companies are registered with the Drug Regulatory Authority of Pakistan (DRAP). The majority of pharmaceutical sales companies are local, domestic companies as opposed to multinational companies [16]. The high-value and powerful pharmaceutical industry generates sales of over US$2 billion per annum, of which two-thirds are prescription drugs [16]. Studies show that almost 90% of medicines in Pakistan are prescribed by their brand names, demonstrating the influence of pharmaceutical companies, and that MSRs are the main information sources regarding medicines for most physicians [28].

Despite national policies to regulate the pharmaceutical industry and its marketing practices, the ‘well-established, symbiotic’ [7] relationship between pharmaceutical companies and physicians, coupled with weak policy implementation, may have turned inducement through MSR into an acceptable norm in Pakistan [16, 29]. An exploratory qualitative study by de Andrade et al. (2018), and a more recent survey conducted by Gul et al. in 2021 (albeit biased in participant selection), provide initial evidence of the pharma-physician relationship and the weak regulatory environment in creating the space for powerful market forces to prevail [16, 29]. Yet detailed empirical information of incentivisation dynamics is still lacking. Building upon initial research, our study systematically characterises the different types of incentives exchanged between physicians and MSRs, and compares incentivisation in practice to the stated key policies on ethical practice. We aim to provide empirical evidence to strengthen the current discourse on unethical exchanges between the pharmaceutical industry’s MSR and physicians in the Pakistan context, and to identify any weaknesses in the formulation or implementation of existing policy documents that contribute towards an enabling environment for these interactions to persist.

## Methods

### Study setting and participants

The mixed-methods study was conducted in Karachi, which has the largest population of medical doctors in the country. We focused on private primary care physicians (i.e., general practitioners) since the majority of healthcare in Pakistan [approximately 74%] is delivered by the for-profit private sector [30]. Further, unlike physicians only working in government facilities, physicians who work in private clinics have more freedom to meet with MSRs and agree to deals with them. There is thus more of a risk that conflicts of interest will occur and, owing to the widespread use of for-profit private providers, improving the quality of care they deliver is vital to overall health system performance. Physicians were considered eligible for the study if they ran a solo for-profit primary care clinic, with or without additional work in a government facility (dual practice). Eligible physicians were initially identified from the research team’s institutional and individual networks, and then purposively sampled to ensure representativeness across Karachi’s six administrative districts and a range of sociodemographic characteristics (Table 1). We additionally explored districts by-foot, asking [eligible] physicians directly if they would consider participating in the study. To identify MSRs, the research team, again, used their own networks and then applied a snowballing technique to widen the selection. Eligible MSRs were those in positions of managers or salespersons at multinational, national, or franchise-based companies.

**Table 1:** Study participant characteristics.

Characteristics	Participants	
	Private primary care physicians	Medical sales representatives
Size of study population	28	12
Age range [years]	26–76	25–38
Gender	Male: 24	Male: 12
	Female: 4	
Location of clinics	Central: 6	MSRs worked across districts
	South, East, Korangi: 5 [each]	
	West: 4	
	Malir: 3	
Type of practice	Single practice: 13	Multinational: 4
	Dual practice: 15	National: 5
		Franchise: 3

### Approach to conducting interviews and data analysis

We conducted semi-structured interviews with the physicians and MSRs from February to July 2021. The interview guide for physicians covered perceptions of the pharma-physician relationship, experience or observations of incentivisation and factors that drive physician-induced demand for medicines. The exploratory interview guide for MSRs covered the extent to which pharmaceutical companies offer incentives (and what types) to physicians, how MSRs build relationships with physicians, and their perceptions about the ethics of incentivising physicians. All bar one interview with a physician was audio-recorded and conducted in the Urdu language. Interview transcripts were translated into English by independent translators and checked for accuracy by bilingual members of the research team (Urdu and English), who read the English transcripts while listening to the Urdu audio to identify and correct any translating errors.

We conducted a thematic analysis of the transcripts. SS and ARS analysed the same ten transcripts inductively (and independently) to develop a codebook. The codebooks were compared and discussed among the wider research team, two of whom tested it independently on approximately five transcripts to reach consensus on the coding. The final codebook organised the data under three major themes: the MSR-physician relationship, types of incentives offered and accepted, and general perspectives on incentivisation. SS and ARS coded each of the remaining transcripts deductively in NVivo using the finalised codebook, such that each transcript was coded independently twice.

### Policy document selection and analysis

We analysed ethical practice policies from the two regulatory bodies responsible for the pharmaceutical industry and medical providers in Pakistan, and the WHO’s *Ethical Criteria for Medicinal Drug Promotion*. Box 1 describes each policy in more detail. We extracted policy statements on incentivisation and broadly classified whether they were prohibitive or permissive; a statement was considered ‘prohibitive’ if it explicitly objected an incentive category and ‘permissive’ if it allowed an incentive category.

**Box 1. Background on three key policy documents analysed in this study**

DRAP functions under the auspices of the Ministry of National Health Services Regulations and Coordination, Government of Pakistan. The agency exercises its powers conferred by the Drug Regulatory of Pakistan Act, 2012, which forms the basis of all DRAP regulations. It is responsible for enforcing the 1976 Drugs Act of Pakistan, thus making DRAP a crucial actor in regulating relationships between pharmaceuticals and medical professionals. The *Regulation to provide Code of Conduct for Ethical Marketing to Health Care Professionals* addresses interactions between health care professionals and pharmaceutical companies from sponsored trainings and educational meetings to gifts and entertainment, educational items, and third party educational conferences [32]. The Code assigns responsibility for its implementation and compliance to pharmaceutical companies’ senior management and does not itself contain any penalties for noncompliance but refers to the penalties contained in the DRAP Act, 2012, under which this Regulation falls. In November 2021, DRAP updated the 2017 Regulations to the *Ethical Marketing to Healthcare Professionals Rules*, *2021* [40]. One marked difference from the 2017 Regulations is a new section on enforcement, which grants regulatory authorities the power to audit pharmaceutical companies.

Until October 2019, the PM&DC was the formal statutory regulatory authority that oversaw medical and dental colleges in Pakistan. However, the PM&DC Ordinance 1962 was replaced by the Pakistan Medical Commission (PMC) Ordinance 2019, making PMC a successor of PM&DC. Although several rules and regulations have changed under the new ordinance, the *Code of Ethics of Practice for Medical and Dental Practitioners*, *Regulations* issued by PM&DC in 2011 has not yet been replaced [31]. The Regulations provide guidance on the practitioners’ code of conduct towards the medical profession and the patient, as well as guidance on interactions between practitioners and pharmaceutical industry including the exchange of gifts, inducements, or promotional aids; use of drug samples; attending meetings, conferences, and hospitality; endorsement of drug or medical equipment; and receiving direct payments from medical research initiatives. While there are no specific provisions for implementation and enforcement of the Regulations, complaints and violations can be brought to the PM&DC to adjudicate. The most severe penalty includes (public) disqualification of the practitioner’s medical license.

The WHO issued guidance on *Ethical Criteria for Medicinal Drug Promotion* in 1988 as a normative framework for the standards of interactions between the pharmaceutical industry and physicians [33]. The ethics criteria described by WHO include promotion in the form of financial or material incentives, advertising in line with scientific evidence and whom to advertise to, roles and responsibilities of MSRs, use and implications of free samples, incentivising medical professional through meetings and symposia. While these guidelines directly address national governments and oblige Member States to adopt the criteria, they are not legally binding. The document acknowledges what is considered ethical ‘varies in different parts of the world and in different societies’ [33] and that the criteria outlined constitute general principles, which governments may adapt to national circumstances. The onus is thus on governments to develop appropriate accountability and enforcement measures.

### Ethics statement

The team received ethics approval the Aga Khan University Ethics Review Committee (reference 2020-4759-1129), the National Bioethics Committee of Pakistan (reference 4-87/NBC-582/21/1364), and the London School of Hygiene and Tropical Medicine Research Ethics Committee. Informed consent was obtained from all study participants in written form.

## Results

Our results synthesise findings from 41 interviews (28 for-profit primary-care physicians and 13 MSRs) and policy statements extracted from the three policy documents analysed that were relevant to incentivisation (approximately 20 in the DRAP *Ethical Marketing to Healthcare Professionals Rules*, *2021*; 23 in the PM&DC *Code of Ethics of Practice for Medical and Dental Practitioners*, *Regulations*, *2011*; and 10 in the WHO *Ethical Criteria for Medicinal Drug Promotion*, *1988*). Table 1 summarises characteristics of the interviewees.

### Incentivisation in practice versus policy

It was clear from the interviews that offering incentives to physicians by MSRs to influence clinical decision-making was the norm; this practice was mentioned in all 40 interviews included in the analysis:

“*As you continue your interviews*, *you will keep getting different views and you will see that 99% of the doctors will be involved*. *I am talking about the doctors who are my colleagues; they do it with the pharmaceutical companies*. *They give air tickets for going abroad*, *give cars*, *and give gifts*.*” [Physician-05]*

“*It is 100% common*. *Every doctor has some kind of arrangement*, *whether it is me or a small doctor or a big doctor or a quack*, *the sale is counted towards him*. *I know this*.*” [Physician- 02]*

We identified five broad categories of incentives, often referred to as ‘activities’ by MSR: financial, material, professional or educational, social or recreational, and familial. Table 2 summarises each incentive category, the number of interviews it was mentioned in, and relevant statements from the three organizations’ policies. While we were able to broadly categorise incentives, certain types of incentives fell under multiple categories depending on the context within which they were offered. For example, holidays and tourism activities could be offered as part of a professional opportunity, or as a purely recreational activity, or a familial activity. We also found incentives could be offered in combination, for example some form of financial incentive (e.g., cash) plus a variety of material and familial incentives, such as gifts and an all-expenses paid trip for the family. In fact, the majority of interviewees described ‘packages’ of incentives and elaborate incentives schemes, which served to increase physicians’ obligation toward the MSR or pharmaceutical company and more deeply entrench their arrangements with MSRs across a number of personal and professional needs. Indeed, data from one MSR highlighted how dynamic incentivisation schemes can be; over the course of the COVID-19 pandemic, he noted that in-person professional or educational opportunities weren’t possible, so the demands and offerings shifted to the provision of infection prevention measures, such as sanitizers, masks, and clinic fumigation.

**Table 2:** Incentive categories and relevant policy statements from the three organizations.

Incentive category	Description	Total # of interviews	Policy statements		
			Drug Regulatory Authority Pakistan^1^	Pakistan Medical and Dental Council^2^	World Health Organization
Financial	Direct and indirect transfer of funds to physicians to increase their wealth.	36 [25 physicians; 11 MSRs]	**Prohibits** companies from providing ‘gifts’ to doctors, which includes cash.	**Prohibits** doctors’ professional autonomy or integrity from being compromised once any ‘gift, benefit in kind or economic advantage’ is offered to them as an inducement. Also discourages doctors from entering into business or other arrangements that include financial incentives.	**Prohibits** financial or material benefits being offered to or sought by doctors to influence their clinical decision-making.
Material	A range of high and low value items that benefit physicians personally and/or professionally.	35 [23 physicians; 12 MSRs]	**Prohibits** companies from providing ‘gifts’, which includes gift cards, food, flowers, or branded promotional items, even if the item is of minimal value. **Permits** companies to occasionally provide items to doctors that benefit patients or serve a genuine educational function for HCPs. Educational items should be modest in cost, as determined by local standards, and should not be provided in excess.	**Permits** doctors to occasionally accept promotional aid items from companies, provided that these items are primarily for the benefit of patients, as well as text or reference-books, medical journals, and other educational materials, if they are satisfied that these serve a genuine, demonstrable and direct educational function.	**Prohibits** financial or material benefits being offered to or sought by doctors to influence their clinical decision-making.
Professional or Educational	Opportunities to acquire new knowledge, develop skills related to the medical profession and expand professional networks.	26 [16 physicians; 10 MSRs]	**Prohibits** companies from directly paying for, or reimbursing, individual doctors to attend conferences; any support must not inappropriately benefit individual doctors or provide for side-benefits e.g., trips, recreation, entertainment or lavish meals or accommodations. **Permits** companies to provide training and education for doctors on company products, at which companies may provide reasonably-priced meals or, when it is impractical or inefficient to provide training at or close to a doctor’s place of business, reasonable travel and accommodation costs.	**Permits** doctors to accept industry support to attend CME or scientific and educational conferences or professional meetings, provided that any financial support is strictly through cheque or bank draft deposited in a duly designated account rather than in their personal bank accounts and that the support is disclosed to the institution and to the Council on demand.	**Prohibits** any support provided to individual doctors to participate in any domestic or international symposia being conditional upon any obligation to promote any medicinal product.
Social or Recreational	Opportunities outside of the professional setting to socialise or to participate in activities that primarily constitute leisure or entertainment.	20 [13 physicians; 7 MSRs]	**Prohibits** companies from providing, organizing or paying for recreational or entertainment activities for doctors, including [without limitation] tours, cultural or artistic activities, or leisure activities. **Permits** MSRs to meet doctors from time to time to discuss products, conduct contract negotiations, or discuss sales terms. Such discussions may take place at another mutually convenient location, provided it is conducive to the business discussion. Meals must be modest and incidental to the business discussion.	**Permits** a maximum of twenty percent of the total time allocated to attending an industry-sponsored event to recreational activities, which are in accordance with the dignity of the medical profession.	**Permits** entertainment or other hospitality, and any gifts offered to doctors, so long as they are secondary to the main purpose of the meeting and kept to a modest level.
Familial	Financial and non-financial benefits aimed primarily at physicians’ families.	15 [9 physicians; 6 MSRs]	**Prohibits** spousal expenses as part of consulting arrangements with doctors.	None	**Prohibits** invitations and financial support to attend industry-sponsored events that include doctors’ spouses or children.

^1^ DRAP statements extracted from the 2017 Regulations.

^2^ Formerly Pakistan Medical and Dental Council

Financial incentives were the most frequently mentioned category of incentives, mentioned by 36 interviewees (90%), and included a variety of direct and indirect means (Box 2). Some of these indirect routes are captured in the quotes given below:

‘What do most of the companies do if authorities come to check if there is an inflow and outflow of cash? Most of the companies give material items [instead]. There is a well-established arrangement of doctors in the electronics market; when a one or one and half ton AC comes [to the doctor] from the company, it will come here and go there [to the electronic market], and they will exchange it for cash from there.’ [MSR-05; edited for clarity]

‘When you get associated with a pharmaceutical company, the doctor also asks which drug store [the MSR] has kept the medicines in, so obviously he will send the patients only there … If the patient buys it from that store, then his [the MSR’s] income will also go up and so will the doctor’s commission.’ [Physician- 05]

**Box 2. Examples of incentives identified**

Financial incentives (direct)

- Cash in-hand
- Cheques
- Bank transfers to physician’s personal accounts
- Illicit payments e.g., kickbacks, for facilitating sales at specific pharmacies

Financial incentives (indirect)

- Gifting physicians with items worth a certain (monetary) amount e.g., electro-cardiogram machines, that they can sell for the equivalent in cash
- Making physicians shareholders of the pharmaceutical company
- Paying physicians maintenance or utility bills e.g., car servicing, air conditioning upkeep

Material incentives (personal)

- Nondescript ‘gifts’
- Food items e.g., sweets, cakes, fruit baskets, traditional breakfasts delivered
- Mobile phones
- Home appliances e.g., LCD screens, generators, fridges
- Cars
- Property e.g., a flat, a bungalow

Material incentives (professional/clinic-related)

- Medical books
- Medical equipment
- Clinic improvements e.g., water dispensers, air conditioning units, LED lighting, furniture
- Clinic renovations e.g., repainting, cleaning, recarpeting

Professional or educational incentives

- Local events e.g., webinars, lectures, seminars, trainings
- International events e.g., conferences, symposia

Social or recreational incentives (offered to physicians alone and/or with their families)

- Dining experiences
- Local and international holidays or tourism activities e.g., tickets to the FIFA World Cup

Familial incentives

- Physicians’ children’s tuition fees
- Physicians’ daughter’s dowry
- Offering to pay for physicians’ family events e.g., weddings, birthdays, anniversaries, and funerals

When comparing practice to policy, all three policy documents that were analysed prohibited financial incentives, though no policy defined nor detailed what would constitute a financial incentive and used vague terms, such as ‘gifts’ in the DRAP policy, ‘economic advantage’ in the PM&DC policy, and ‘financial benefits’ in the WHO policy, which could refer to a variety of different direct and/or indirect financial incentive types.

Material incentives were the second most frequently mentioned category, mentioned by 35 (88%) interviewees. The types of incentives identified varied in size and value, as summarised in Box 2, and included numerous incentives to enhance clinics. As one physician, explained, the offerings are substantial but not without linked targets for prescribing:

‘*In this one year*, *I have been offered a car also*. *But they have conditions… that within one month you have to achieve the target*.*’ [Physician-03]*

Both the DRAP and PM&DC policies allow for some flexibility in providing or accepting material incentives, as long as, according to DRAP, they ‘serve a genuine educational function’, are ‘modest in cost’, and not ‘provided in excess’, or, according to PM&DC, are ‘primarily for the benefit of patients’ [31, 32]. Any number of incentives identified in our research could be justified by these policies and, indeed, this was a rationale used by a number of interviewees as illustrated below:

‘*But to achieve the specific [sales] target*, *the company has not allowed offering cash*, *but the company has said this*, *that if you give the doctor an ECG machine then he can do a good diagnosis*.*’ [MSR-08]*

‘*They get the whole clinic renovated*. *Everything comes [with] it*, *from 0 to 100*: *it includes repainting it*, *there is work on the doors*, *or the furniture*.. *the needs of the patient in the waiting area [of the clinic] are fulfilled*.*’ [MSR-09; edited for clarity]*

Professional or educational incentives were mentioned by 26 (65%) interviewees. When discussing this incentive category, interviewees frequently talked about the side-benefits commonly offered as part of or alongside the educational opportunity, such as travel fare, accommodation, meals, and various forms of entertainment or leisure activities. As one interviewee expressed, ‘*70% [of doctors] go for entertainment*, *30% gain some knowledge*’ [Physician-01]. We found that educational events can be used to oblige physicians in different ways: providing access to the educational opportunity itself is used by pharmaceutical companies to induce prescriptions of their products, or the variety of side-benefits accompanying the educational opportunity (e.g., first-class airfare, five-star accommodation, luxury dining experiences, cultural tours and activities), can result in an obligation to prescribe the sponsoring company’s medicines. Here the policy documents are not comprehensive in covering the routes of incentivisation by pharmaceutical companies through educational events (Table 2). The WHO policy prohibits any support that is conditional upon the promotion of products, while the DRAP policy prohibits any support from ‘inappropriately benefiting’ individual physicians (including side-benefits) but falls short of addressing sales targets set by pharmaceutical companies in exchange for attending the educational event. Under the DRAP policy, companies are allowed to provide training and education to physicians along with ‘reasonably-priced’ travel, meals, and accommodation [32]. The PM&DC policy adopts a generally permissive statement, allowing physicians to accept support from pharmaceutical companies, even allowing pharma-issued cheques or bank transfers to ‘duly designated’, not personal, accounts [31].

‘*There will be a lecture by the company for a couple of hours in those five days and the rest you do sightseeing*, *etc*., *as the company has to show that they are taking [doctors] for CME*.*’ [Physician- 01]*

‘*If my [company name redacted] product is sold a lot*, *like if my sale is two*, *three*, *five*, *eight lakhs per year*, *then [the MSR] will be able to give me a CME of one and a half lakh rupees*. *Like he will be able to send me to a conference in Italy or Cancun*.*’ [Physician- 02]*

Social or recreational incentives and familial incentives were less frequently talked about in interviews, being mentioned by 20 (50%) and 15 (38%) of interviewees respectively. The PM&DC and WHO policies are generally permissive of social and recreational activities within the context of a professional or educational event, only introducing arbitrary limits such as a maximum time allocation, being ’secondary’ to the main purpose of the event, and kept to a ‘modest’ level [31, 33]. Furthermore, these policies do not call out the common practices we identified outside of professional or educational activities, such as paying for physicians’ meals or holidays. The DRAP policy begins by prohibiting this form of incentivisation but then allows MSRs to meet with physicians in non-professional settings and provide meals [32]. In the familial incentives category, we identified a number of highly-personalised examples (Box 2). There was limited coverage of these types of incentives in the policy documents, and those policies with statements (DRAP and WHO) only addressed familial incentives within the context of another incentive category, such as a business contract or a professional/educational event. They missed a number of intricate and unusual incentives in this category that we were able to identify, which further entrench the MSR into the physicians’ personal lives.

‘*So [MSRs] say [to doctors] that we know your daughter is taking pre-medical [examination for] getting admission for an MBBS degree and we have heard she is quite good*, *but she couldn’t get admission in [a public college]*. *But if you want to get her into a [higher fee] private college*, *then we can support*.*’ [MSR-08]*

‘*But if you see today*, *a father’s 40 day of death [prayer ceremony] is being paid for by [a] pharmaceutical company*, *walima [wedding reception] is being paid for by the pharmaceutical company*, *and this is the state of our country*.*’ [Physician- 02]*

### How MSRs initiate, build, and sustain relationships with physicians

The interviews with the MSRs revealed a highly strategic process that MSRs follow to select which physicians to approach and which to initiate a relationship with. Some MSRs described a classification system used to rank physicians according to how many patients they routinely see, which determined their ‘business potential’ and priority for MSR. A number of physicians also noticed this trend, as one interviewee stated, ‘*pharma people approach the ones [physicians] who have heavy practice*’ [Physician-02]. The interaction between the MSR and the physician informed the frequency of the MSRs’ visits, how and when they would broach the topic of incentives, and the types of incentives they offered to the physician. According to some MSRs, a regular time investment, for example paying physicians frequent visits, was sufficient to convince them to prescribe their products while other physicians would not change their prescription practices until an incentive is offered. If physicians refused incentives or did not make their sales targets, both MSRs and physicians explained that they would not be approached again, and a relationship would not develop.

‘*The way is that these pharmaceutical companies are also in touch with each other about doctors*. *Whoever comes to me now has understood my nature*.*’ [Physician-03]*

‘*What happens is that the “A class” doctors… they resist having any irrelevant discussions with them*. *If we look at “B class” doctors*, *they have a friendly nature*…*So*, *the “B class” doctor is not only friendly*, *but he is also not resisting this kind of discussion [about incentives]*.*’ [MSR-05]*

‘*[Sometimes] we get our medicines prescribed on footwork meaning we go on daily routine and meet them*. *Doctors feel good that he has come*, *and I will write it because of him*. *Second one is*, *even though we are meeting*, *until we give cash*, *he is not going to write it*.’ *[MSR-09]*

Physicians also appeared to have some criteria for evaluating whether an MSR was worth their time, for example a number of physicians said they would only accept incentives from MSRs from ‘good companies’. This was based on their perceptions of the pharmaceutical company’s reputation, the marketed drug’s efficacy [according to the MSR], and its price point. However, one MSR interviewee suggested that if a physician has a strong relationship with a MSR, they could be convinced to prescribe that MSR’s brand regardless of the drug’s perceived efficacy. Once MSRs had established a relationship with a physician, they identified their needs, either personal or professional (or both), to determine what types of incentives to offer them. The MSRs we interviewed indicated that they would often do this by asking the physicians directly what they desired. After learning what types of incentives were most attractive to the physician, MSRs communicated this to more senior company employees who would approve the incentive(s). Two MSR interviewees alluded to a level of confidentiality or a ‘*contract*’ [MSR-04], between the pharma company, the MSR, and the physician regarding the incentives or ‘activities’ offered. The legal nature and details of such a contract was not disclosed by the interviewees.

‘*It depends upon the need*. *If the doctor needs cash*, *then 20% is in the form of cash*. *If their need is to get a tour*, *then it can be in the form of tour*. *If the need is for planning anniversary*, *then it will be in that form*. *So as per the need*, *the doctor is going to tell you*.*’ [MSR-04]*

‘*The doctors can ask anything… And whatever amount of money they give to the doctors*, *then to such ratio the doctor will give them business*.*’ [MSR-08]*

Several MSR and physician interviewees reported that multinational companies (MNCs) had stricter limits on what could be offered compared to national companies; for example, MNCs prohibit any form of direct financial incentive. However, interviewees also stated that this did not preclude MSRs from MNCs offering financial incentives in indirect and covert ways, along with a variety of other incentive categories. An example of this described by interviewees was the ‘gifting’ of items, such as air-conditioning units or electro-cardiogram machines, with the tacit understanding that physicians would sell these items for the equivalent in cash. Or alternatively, they offer more attractive international professional and educational opportunities. Some MSRs, regardless of whether they worked for a MNC or a national company, were also reported to act somewhat independently of their companies’ policies, taking decisions regarding incentives at their own discretion from personal funds—often to advance their own agendas and meet more ambitious targets, which would result in greater bonuses at the end of the month.

Interviewees had mixed opinions on who—pharmaceutical companies/MSRs or physicians—were ultimately responsible for driving the relationship dynamics. On one hand, several physicians felt very strongly that they were the victims in this cycle and had been increasingly corrupted by the pharmaceutical industry, which held more power and resources. On the other, a minority of physicians and several MSR criticized the medical community for encouraging an incentives-based culture; both physician and MSR interviewees reported the active role that some physicians play in driving incentivisation by demanding any incentive(s) they want. These interviewees thought that physicians should take more responsibility for their part in sustaining the culture. Both physician and MSR interviewees widely described the relationship as ‘*give and take*’, based on a shared understanding of the stakes and the mutual benefits involved.

## Discussion

Our study provides detailed empirical evidence on the dynamics of incentivisation between for-profit physicians and pharmaceutical companies in Pakistan. It is the first study, to our knowledge, that systematically characterises the extensive range of incentives exchanged via MSRs, and documents how widespread incentivisation linked to prescription targets is. It also provides new evidence on MSR’s strategic categorisation of physicians according to their ‘business potential’ and their personal needs. Additionally, our findings show that some physicians play an active role in asking MSRs for specific types of incentives, indicating that there is a two-way dynamic to this relationship and an underlying symbiosis driving incentivisation.

Consistent with our findings, studies in other contexts have reported marketing strategies whereby pharmaceutical companies profile physicians to evaluate and better cater to their needs [34, 35]. For instance, in Bangladesh, a well-known multinational company categorized doctors into ‘types’ based on what the doctor values, such as the ‘Affiliating type’ who welcome MSRs and are easier to work with compared to the ‘Directing type’, for example, who show no interest in incentives [36]. The symbiotic relationship between physicians and the pharmaceutical industry that our findings highlighted has also been found in recent studies in the United States and France [35, 37, 38].

Incentivisation is a challenging issue to study owing to the inherent conflicts of interest and sensitivities surrounding the ethics of a physician’s professional conduct. We thus acknowledge the limitations of our qualitative findings in terms of the extent to which interviewees might have revealed the full reality of incentivisation in their own practice. Nevertheless, we found interviewees to be forthcoming in sharing their perspectives and experiences, which might have resulted from some of the techniques we used; for example, we asked questions about incentivisation indirectly and used vignettes to allow interviewees to talk about incentivisation practices in hypothetical scenarios, rather than discussing their own personal experiences and details of deals they might have with MSRs.

Another limitation is that our study sample contained a small number of female physicians, which reflects the composition of the private primary healthcare sector in our study setting [39]. We therefore could not analyse gender differences in attitudes towards incentivisation. Similarly, although we included participants who worked in government facilities as well as in the private sector [dual practice] our study did not aim to compare attitudes between dual practice and private sector only physicians; this may be an area for future research. Our initial findings indicated that dual practice physicians were more open to answering sensitive questions around the incentive-types generally exchanged, as they portrayed themselves being more financially stable and ethical owing to their government jobs.

Further to the interviews, our comparison of incentivisation practices with key policy documents identified specific weaknesses in how policies are formulated and implemented that contribute toward an enabling environment. First, some clearly stated policies were being ignored by pharmaceutical companies and physicians; for example, taking financial incentives with linked prescription targets is not permitted according to PM&DC, DRAP and WHO policies and yet, it is a widespread practice. This level of noncompliance suggests that, firstly, there is weak enforcement of the policies, specifically the PM&DC and 2017 DRAP policies given the WHO guidelines are not legally-binding on Member States (Box 1); and secondly, that the consequences for noncompliance outlined in these policies are not sufficient to deter deviant behaviour. It is premature at this stage to suggest how (if at all) the 2021 DRAP policy update, and the new provisions for enforcement included therein, will impact compliance. Second, we found that the three policies differed in terms of whether they prohibited or permitted–or the extent to which they prohibited / permitted–certain incentive categories. Even within an organisation’s policy, we found a mix of prohibitive and permissive statements on categories such as material incentives, educational incentives, and social or recreational incentives. This was observed particularly within the 2017 DRAP’s policy statements, though the latest update offers some clarity, for example by providing a definition of ‘gifts’ that recognises their ‘tangible or intangible in nature’ and their inherent monetary value [39]. But while the revisions now prohibit the exchange of gifts ‘in any shape whatsoever’, the policy remains permissive of providing certain ‘educational items’ to physicians [39]. These contradictions create the space for flexible interpretation or inconsistent adoption of the policy depending on the incentive category, rather than on the underpinning ethical principles. Finally, some broad categories of incentives are largely unaddressed across all policies (e.g., familial incentives), and some specific incentive types, which are commonly exchanged (e.g., clinic improvements), are not explicitly mentioned within the broader category of incentives.

It is important to reiterate from our findings that even though policy statements were overall prohibitive with regards to financial incentives, this was the most common category of incentives identified in our study, indicating routine noncompliance by pharmaceutical companies, MSRs and physicians alike. While there is some evidence to suggest variable levels of awareness of policies [16], a gap between rules as written on paper and as implemented in practice is well documented in different settings [5, 18, 40, 41]. Therefore, although more explicit and detailed policy positions with linked awareness may be useful, this may not result in widespread changes in practices, given the prevalence of financial incentive exchanges [15, 26, 32, 33]. Here, our study illuminates challenges to implementing regulations in the face of potential widespread opposition from pharmaceutical companies and for-profit healthcare providers, given the win-win of their reciprocal incentives-based relationship [26]. Future research should consider how socioeconomic and structural factors underpin behaviour, to inform regulatory strategies with a fuller appreciation of conflicts of interests [6, 22]. Furthermore, when international guidelines, such as the WHO’s *Ethical Criteria for Medicinal Drug Promotion*, allow some level of adaptation to accommodate national circumstances, it raises questions warranting further investigation on the extent to which ethical medical practice standards are determined by context, rather than by universally applicable principles, such as beneficence and non-maleficence.

## Conclusion

In light of how widespread and entrenched incentivisation has become, we draw two conclusions and propose policy-oriented solutions to move forward on ethical medical practice. First, the incentives culture has evolved beyond what is covered unambiguously in existing policy documents, becoming more elaborate and woven into physicians’ professional and private lives. As such, there is a need for policies—some outdated by more than two decades—to be updated to reflect the current realities. Second, transgressions on target-driven prescribing need to be seen as unethical by pharmaceutical industries and physicians alike. Hence, in conjunction, policies that attract buy-in from pharmaceutical companies and physicians are critical. Failure to gain buy-in for voluntary compliance from powerful physicians and pharmaceutical companies can result in the need for levels of enforcement that are impractical for regulatory bodies, which, in many settings, are significantly under-resourced; and the need to continuously expanding policy guidance to cover the new ways of breaking rules.

## Supporting information

S1 Data Illustrative quotes. In the S1 Data, interviewee codes correspond to the participant (either ‘GP’ i.e., physician or ‘MSR’) followed by their number in the interview sequence and the date the interview was conducted. For the physician interviews, the ‘GP’ in the code is followed by the first letter of the district in which the physician’s clinic is located. For example, GPE-004 refers to a physician in the East district of Karachi, who was fourth in the interview sequence. The MSR interviews did not have a district location and are thus simply followed by their number in the sequence.
(XLSX)
